# Computational Modeling of Claudin Structure and Function

**DOI:** 10.3390/ijms21030742

**Published:** 2020-01-23

**Authors:** Shadi Fuladi, Ridaka-Wal Jannat, Le Shen, Christopher R. Weber, Fatemeh Khalili-Araghi

**Affiliations:** 1 Department of Physics, University of Illinois at Chicago, Chicago, IL 60607, USA; sfulad2@uic.edu (S.F.); rjanna2@uic.edu (R.-W.J.); 2 Department of Pathology, University of Chicago, Chicago, IL 60637, USA; leshen@uchicago.edu; 3 Department of Surgery, University of Chicago, Chicago, IL 60637, USA

**Keywords:** claudin, molecular dynamics, tight junction, ion transport, ion channel

## Abstract

Tight junctions form a barrier to control passive transport of ions and small molecules across epithelia and endothelia. In addition to forming a barrier, some of claudins control transport properties of tight junctions by forming charge- and size-selective ion channels. It has been suggested claudin monomers can form or incorporate into tight junction strands to form channels. Resolving the crystallographic structure of several claudins in recent years has provided an opportunity to examine structural basis of claudins in tight junctions. Computational and theoretical modeling relying on atomic description of the pore have contributed significantly to our understanding of claudin pores and paracellular transport. In this paper, we review recent computational and mathematical modeling of claudin barrier function. We focus on dynamic modeling of global epithelial barrier function as a function of claudin pores and molecular dynamics studies of claudins leading to a functional model of claudin channels.

## 1. Introduction to Tight Junctions

Tight junctions form paracellular barriers between cells. The barriers are established by interactions between transmembrane tight junction proteins located within the apical junctional complex. The regions of interaction were first appreciated ultrastructurally and it was determined that they form a barrier to paracellular flux [1]. In the years since these early observations, we now understand that members of the claudin family of transmembrane proteins are major components of these strands and are important contributors to paracellular charge- and size-selectivity. Extensive data supports that interactions between claudin proteins on adjacent cells are important for tight junction permeability. Understanding how claudins create such charge- and size-selective barriers has been a major research focus. Experiments in combination with mathematical and computational modeling have contributed to understanding the role of claudins in defining and regulating this paracellular barrier. Here, we will provide an overview of the theoretical and computational modeling of claudin pores in recent years and discuss how they have contributed to our understanding of tight junction function. Specifically, we will address what is understood about the dynamic claudin barrier, structural characteristics of claudin pores and the nature of ion selectivity and the ability to self-assemble into multimers (strands).

## 2. Resistive Models of Paracellular Flux through the Tight Junctions

Using transmission electron microscopy, tight junctions (TJ) are appreciated as a region of close apposition of the apical intercellular membranes [1]. Within this region, the membranes appear to be fused at multiple points. Freeze fracture electron microscopy (FFEM), a technique which allows the membrane to be fractured between the two leaflets of the lipid bilayers, permits visualization of the detailed structures within the tight junction. On the outer leaflet, the tight junction appears to contain multiple interconnecting ridges and, on the inner leaflet, the tight junction appears to contain multiple interconnecting grooves [2,3]. These results lead to the interpretation that the tight junction contains multiple interconnecting strands. Within different epithelia, strand numbers vary. Some epithelia have one or two strands, whereas other epithelia have eight strands on average [3]. Claude et al. hypothesized that the strand number may be related to the tightness of the tight junction [4]. If TJ strands act as series of resistors, then there would be a linear relationship between number of strands and transepithelial junctional resistance (since resistors sum in series). However, Claude observed an exponential relationship between strand number and barrier function. To explain this nonlinear behavior of resistive barrier generated by multiple strands, Claude developed a more complex model and hypothesized that strands are populated by “pores” that can open or close to regulate TJ ion conductance [4]. This is the first model to predict the tight junction barrier as a dynamic structure, however we now know such a model is an oversimplification since, even in epithelia with the same number of tight junction strands, the expression of certain claudin proteins can dramatically influence transepithelial electrical resistance (TER) [5,6]. Thus, tight junction barrier is far more complex than originally predicted by Claude et al. [4].

## 3. Dynamic Models of Claudin Function

**Figure 1:**
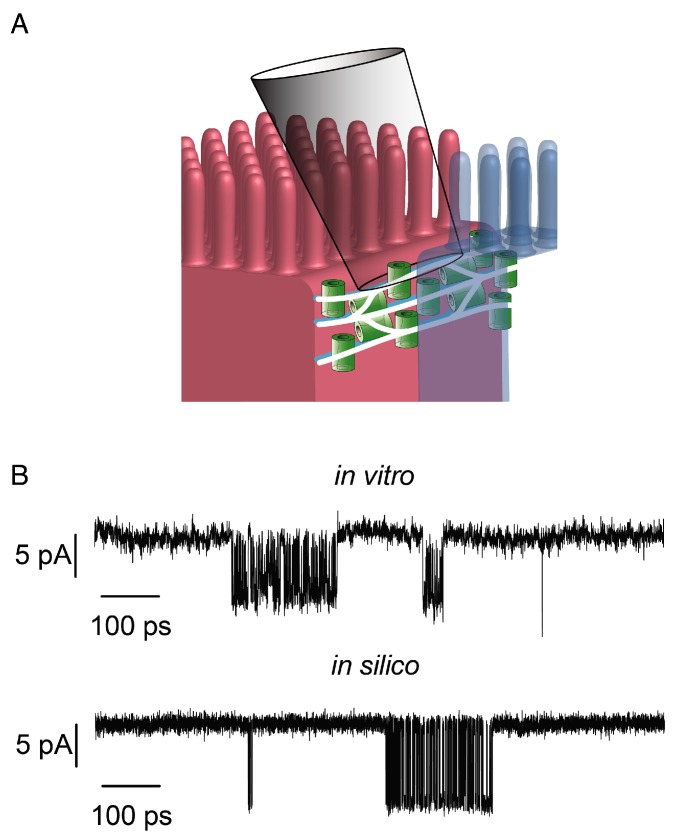
Mathematical modeling of tight junction permeability. (**A**) Electrodes were sealed over the intracellular space between MDCK I induced to express claudin-2 [12]. Each claudin-2 pore was defined by two closed states (C_1_, C_2_) and a single open state (O) according to the equation in [14] (**B**) In silico patch clamp recordings resembled in vitro tight junction patch clamp recordings from MDCK I monolayers expressing claudin-2 (recording from dataset of Weber et al. [12]). For the simulation, each green pore shown in panel A was modeled as 36 resistors per micron with defined opening and closing probabilities [14].

We now understand certain claudin proteins (e.g., claudin-2, -15, -10a, -10b) can selectively increase paracellular permeability of small molecules (<∼6 Å diameter) in a charge-selective manner [7,8,9,10,11]. Since such charge- and size-selectivity could theoretically be explained by insertion of ion channels, this has been termed the “pore” pathway, which is in contrast to large pathways of non-selective conductance termed the “leak” pathway. Because of the hypothesis that tight junctions are populated by dynamic pores, Weber et al. adapted patch clamp technique to study individual claudin-2-dependent pores (Figure 1A) [12]. Comparison of patch clamp recordings of low versus high expressing claudin-2 monolayers revealed a distinct population of dynamic ion channel openings over tight junctions, which were absent from recordings made on the plasma membrane away from tight junctions. These channel openings demonstrated charge- and size-selectivity which reflected globally measured barrier function. The openings occurred during burst-like intervals in which the channels flickered between open and closed rapidly (sub millisecond flickering) with prolonged periods of closure between bursts (Figure 1B). These opening and closing events were strictly due to claudin-2 since mutating one residue within the claudin-2 channel (I66) to a cysteine, allows linkage of the I66C to a bulky molecule (MTSET), through sulfhydryl modification [13]. This modification rapidly and specifically blocked the opening and closing events indicating that the channels can be blocked like conventional transmembrane ion channels [12].

To further understand such dynamic claudin-2 opening behavior, an in silico resistive multistrand model was developed. Claudin-2 channels were arranged within a network of three branching tight junction strands (as observed by FFEM). The channels were allowed to randomly vary between high resistance closed states (10 pS) and low resistance open states (222 pS) with fixed probabilities defined by the patch clamp recordings. Increasing the density of claudin pores from 6 to 36 (closed and open) per strand micron in each of the parallel strands recapitulated tight junction patch clamp recordings in MDCK monolayers with low and high claudin expression (Figure 1B). However, on its own, the dynamic pore pathway could not account for all of the claudin-2 dependent barrier function, as measured by global TER measurements in epithelia monolayers. To make the data fit globally determined TER values, it was necessary to add a parallel leak component. Such a leak component could be due to a steady state conductive component, rare highly conductive openings or high conductance at tricellular junctions which are not readily detectable using tight junction patch clamp recordings. Thus, while the model explains local pore function well, it cannot fully explain leak pathway conductance [12,14]. More complex models to describe leak pathway flux have been postulated. These include both leak through a tricellulin-dependent pathway and leak through breaks in tight junction strands [15,16,17,18]. These studies are beyond the scope of the present review. Thus, patch clamp recordings and modeling suggest the presence of gated ion selective claudin ion channels. However, these studies fail to define the atomic details of the pore, mechanisms of channel selectivity or mechanisms of gating. More detailed studies and atomic-level models are needed.

## 4. A Structural Model of Claudin Pores

A recently resolved crystal structure of mouse claudin-15 provides the first snapshot of a claudin monomer with atomic resolution [19]. The claudin-15 monomer is made of four transmembrane (TM) helices and two extracellular segments ECS1 and ECS2. The two extracellular segments as well as TM helices are known to mediate polymerization of claudins through side-to-side (*cis-*) interactions as well as head-to-head (*trans-*) interactions of claudins in plasma membrane. However, the crystal structure does not contain key portions of the extracellular segments and does not consider interactions between adjacent cells, making it impossible to define the channel structure.

**Figure 2:**
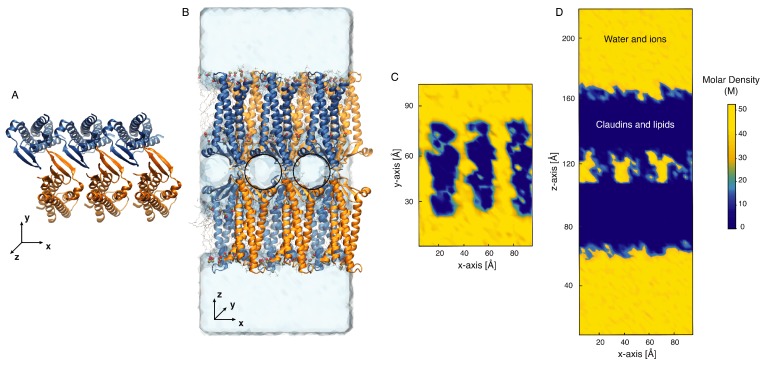
A refined model of claudin-15 paracellular channels [25]. Claudin-15 monomers assemble into double-row strands and form paracellular channels. (**A**) Top view of six claudin-15 monomers assembling into a double-row strand. (**B**) Snapshot of the simulation system showing claudin-15 monomers assembled between adjacent lipid bilayers. The pore regions are marked with circles. The water density across the simulation system and averaged over ∼250 ns of MD simulations, is shown: (**C**) parallel to the two membranes crossing the pores in the middle and (**D**) normal to the two membranes and crossing the pores in the middle. Figure is taken from [25] with slight modification.

Soon after resolving the crystal structure of claudin-15, Suzuki et al. [20] proposed a model of paracellular pores based on the crystallographic arrangement of claudin-15 as well as cysteine cross-linking experiments obtained from mutants of claudin-15 and claudin-2 [13,21,22] and strand dimensions observed in freeze-fracture electron microscopy images [20]. In this model, claudin pores are formed by association of two anti-parallel double rows of claudins in the membrane of two adjacent cells, forming β-barrel like channels parallel to plasma membrane (Figure 2A,B).

The stability of this model was investigated in all-atom molecular dynamics (MD) simulations of claudin-15 molecules in their natural environment of lipid bilayers. Simulations of a single- [23] and a double-pore model [23,24] and a periodic strand model of three pores [25] proved that the proposed architecture of Suzuki et al. [20] creates a stable arrangement of claudin channels. During the 200–300 ns simulation trajectories, *cis-* and *trans-*interactions between claudins in the lipid bilayers are maintained and the β-barrel like scaffold of the pore is preserved. However, simulations of a four-pore model of claudin-15 indicate that there might be multiple and alternative interfaces not captured in this arrangement [24].

In this model, the linear arrangement of claudins in the membrane is maintained through side-by-side (*cis-*) interactions between adjacent claudins involving residues S67 and M68 on extracellular helix (ECH) and residues F146 and F147 on TM3 and E157 and L158 on ECS2 domains. Moreover, the double-row arrangement of claudins in the membrane, as was suggested for the first time by Suzuki et al. [20], was maintained through hydrogen bonds between β-sheets of adjacent claudins. In addition, simulations identified two distinct head-to-head (*trans-*) interactions between claudins in two opposing membranes, residues 39–42 on two opposing ECS1 loops and 146–155 on the ECS2 of two claudins form strong hydrophobic interactions that were maintained throughout the simulations [23,25]. Simulation trajectories show that claudins form well-defined pores in paracellular space that are filled with water and ions. Each pore runs parallel to the membranes and is made of eight claudin monomers. The pores are approximately 50 Å long with a minimum diameter of 5–6 Å [23,25]. The claudin-15 pore is anisotropic in the two directions orthogonal to permeation pathway. Spatial distribution of claudin-15 pores in these simulations suggests that this model corresponds to a density of 300 pores per μm in TJ strands as shown in Figure 2C [25].

Analysis of the water density from simulations of Samanta et al. [25] indicates that water molecules, and thus ions, are confined to well-defined pathways within claudin-15 pores (Figure 2C,D). Water molecules occupy the vestibules formed by claudin pores with a density very close to the bulk density of water. The paracellular space between claudin pores is completely sealed to water molecules. Simulations show that *trans-*interactions between opposing residues (39–42 in claudin-15) on ECS1 (hydrophobic patches) are essential to seal the paracellular space *between* neighboring claudin pores. This is consistent with previous studies suggesting that claudin-15 form water channels [26] and that water and ions are transported through a similar pathway in claudin-2 and claudin-15 pores [26,27].

Based on these refined models of claudin paracellular channels, the underlying mechanism for ion transport through the pores has been studied further.

## 5. Ion Transport Simulations

All-atom molecular dynamics (MD) simulations studies have been very successful in describing functional mechanisms of membrane proteins, particularly ion channels [28,29,30,31]. Having an atomic model of claudin pores provides a unique opportunity to test hypotheses about the structural basis of charge- and size-selectivity in claudin pores. Using MD simulations Samanta et al. [25] investigated the transport properties of claudin-15 pores in a refined model based on the proposed architecture of Suzuki et al. [20]. Transport of ions through the pores was simulated using all-atom MD simulations of three claudin pores in two parallel lipid bilayers. Ionic currents under an external voltage-bias [32] were calculated from the displacement charge associated with all ionic species over the course of simulations resulting in current-voltage relationships and enabling them to estimate the permeability of claudin pores to each ion independently.

Permeability of the pore to monovalent cations of different sizes, Na^+^, methylammonium (MA^+^), ethylammonium (EA^+^), tetramethylammonium (TMA^+^) and tetraethylammonium (TEA^+^) as well as Cl^−^ was calculated. The simulations indicate that claudin-15 pores are cation-selective, with a relative permeability of 4.2 for Na^+^ with respect to Cl^−^. Moreover, simulations suggest that the pore permeability to cations decreases as their size increases. While the claudin-15 pore is permeable to small cations such as MA^+^, EA^+^ or TMA^+^, transport of larger cations such as TEA^+^ (radius 3.5 Å) through the pore is significantly hampered by their interaction with the pore surface. This is consistent with previous experiments and Brownian dynamics simulations of the cation-selective claudin-2 pore, which suggest a minimum pore radius of 3.25 Å [9].

Trajectories of ion transport obtained from the simulations showed that transport pathways of cations and anions in claudin-15 are different. Cl^−^ ions interact minimally with the pore surface and pass through the middle of the pore, while cations such as Na^+^ slide along the inner surface of the pore and interact strongly with negatively charged amino acids on the pore surface. Simulations identified four binding sites for cations inside the pore. These binding sites are located in the middle of the pore and are mainly formed by sidechains of D55. One, two or three Na^+^ ions were observed to bind to D55 residues simultaneously, while larger cations such as TEA^+^ bind in the middle of four D55 residues. To further quantify the charge-selectivity mechanism of claudin-15, Samanta et al. [25] calculated the contact time of the permeating ions with the amino acids lining the pore surface. The peak interacting site for cations was at D55, while negatively charged residues within the pore (E46, D64, D145 and E157) showed significant but much shorter interaction time with cations. Analysis of the contact map of claudin-15 suggests that D55 is the key residue controlling the charge-selectivity of claudin-15 in this model.

To define the role of D55 and other residues of claudin-15, Samanta et al. simulated conductance of several claudin-15 pore mutants [25]. Mutation of D55 to a neutral amino acid (D55N) decreased the charge- (cation) selectivity of the pore and mutation of D55 to a positively charged amino acid (D55K) reversed the charge-selectivity of claudin-15. Moreover, D64K and E46K mutations decreased the cation-selectivity of the pore. However, the triple mutation of E46K/D55K/D64K, similar to D55K mutant reversed the charge-selectivity of claudin-15 resulting in anion-selective pores. These findings were supported by in vitro measurements of transepithelial resistance (TER) of claudin-15 and its mutants in MDCK I monolayers expressing claudin-15. However, one feature that differed from the modeling is that the triple mutant (E46K/D55K/D64K) was not strongly anion-selective in vitro. We speculate that this may relate to the expression of other tight junction proteins in vitro.

In a more recent study, Alberini et al. [33] calculated the potential of mean force (PMF) for the ion permeation through claudin-15. The PMF along the ion permeation pathway provides a quantitative measure of the pore selectivity by quantifying the free energy barriers/wells that each ion encounters as it passes through the pore. The simulations were performed on an equilibrated configuration of a single-pore model of claudin-15 [23] based on the proposed architecture of Suzuki et al. [20]. The free energy profiles were calculated for Na^+^, K^+^ and Cl^−^ along the central line of the pore from 325 ns of equilibrium simulation using the umbrella sampling method [34]. The free energy profiles show a free energy barrier of 8 kcal/mol (13.5 k${}_{B}$T at room temperature) for Cl^−^ ions and an attractive well of approximately 4 kcal/mol (6.7 k${}_{B}$T) for Na^+^ and K^+^. There was no significant difference between the free energy profiles of Na^+^ and K^+^ in this case. A barrier of 8 kcal/mol is high enough to prevent passage of Cl^−^ ions through the pore at room temperature, and an attractive well of 4 kcal/mol is strong enough to attract cations while at the same time allowing them to pass through the pore at room temperature. Interestingly, the position of the free energy barrier/well in the PMF corresponds to the location of D55 residues in claudin-15.

To study the kinetics of ions through the pore, Alberini et al. used the Voronoi-tessellated milestoning method [35,36] to calculate the transport rate of ions through claudin-15 and determined a ratio of 25 to 1 for the transport rates of Na^+^ with respect to Cl^−^ and a ratio of 1 to 1.4 for Na^+^ with respect to K^+^. It has to be noted that transport rates calculated from free energy profiles usually have large error bars due to the limited accuracy of the force fields and the inherent errors in the free energy calculations. However, in a recent experimental study, the relative permeability of claudin-15 for Na^+^ and K^+^ was estimated to be close to 1 [26]. Simulations of Alberini et al. [33] showed that cations become partially dehydrated as they pass through claudin pores. The narrowest region of the pore is approximately 6 Å in diameter, while the diameter of a fully hydrated Na^+^ ion is ∼ 7Å. Further analysis of the hydration state of Na^+^ ions in these simulations showed that Na^+^ ions are coordinated by approximately 5.7 oxygen atoms throughout the simulation. At the entrance of the pore all these oxygens are contributed by water molecules. However, as the cation moves along the pore, interaction with protein side chains temporarily replaces the interaction with water molecules. In particular, as the ions approach D55 in the middle of the pore, one or two of the oxygen atoms within water molecules are replaced by oxygen atoms of the D55 residues, consistent with favorable interactions between protein and cations at this location.

Simulations of ion transport show that the putative architecture of Suzuki et al. [20] is a viable model of claudin pores that is energetically stable and describes the nature of ion selectivity in TJs. However, this model of claudin-15 assembly does not conclusively explain all of the experimentally obtained results across various claudins. In particular, this model is not compatible with studies suggesting the role of TM helices in claudin assembly and strand formation through *cis-*interactions [37,38,39,40,41]. Based on these interactions, alternative pore models have been proposed [37,42,43]. With recent advances in computational techniques, molecular dynamics simulations can reach time scales and sizes relevant to study assembly and initial stages of protein aggregation [44,45,46,47,48,49] and can provide an insight into alternative conformations of claudins in the TJ strands.

## 6. Claudin Polymerization in Lipid Bilayers

Coarse-graining (CG) methods complement atomistic MD simulations and enable researchers to study larger biological systems over longer time scales. In particular, CG modeling is used to study membrane protein self-assembly [47,48,49]. These approaches have been used to understand how claudin monomers may interact within TJ strands (reviewed in [50]).

Using CG methodologies [51], Irudayanathan et al. [52] studied the self-assembly process of a homology model of claudin-5 in a lipid bilayer. The authors studied two systems of uniformly distributed claudin-5 monomers and simulated self-assembly of these monomers into multimeric units. These simulations revealed rapid association of claudin-5 into dimeric structures, which later aggregated into strands of 16 to 36 claudin-5 molecules within 10 μs. The CG simulations predicted four prevalent interfaces between claudin-5 monomers within a lipid bilayer. Two of these dimeric structures are compatible with the *cis-*interactions proposed in earlier models [20,53], one between adjacent claudin monomers in a row (side-to-side) as observed in the crystal structure of claudin-15 and the other, between β-sheets of claudin molecules from opposing rows (face-to-face). These two interaction surfaces are consistent with the proposed model of Suzuki et al. [20]. A third dimeric interaction was between TM helices (TM2–TM3) of two claudins. This claudin-5 dimeric interface is consistent with previous studies suggesting the close vicinity of TM2 and TM3 helices between claudins [38,39]. A fourth dimeric structure involves interactions between TM helices and ECSs (TM3–ECS2–TM4) of two adjacent monomers. The role of TM3 and TM4 was shown previously using alanine-insertion mutagenesis (AIM) analysis of claudin-16 and claudin-19 heterodimers [41]. These CG simulations were extended to investigate assembly of homology models of other claudins, claudin-1, -2, -4, -15, and -19 in single lipid bilayers and the same four dimeric interfaces were observed [54].

To assess the relative stability of the observed dimers, Iruyadanathan et al. constructed atomic models of the four dimers of claudin-5 observed in CG simulations and calculated the binding free energy (PMF) of claudin-5 monomers in each configuration [52]. Their results show that dimeric conformations that involve more interactions sites through TM helices are more stable compared to dimers involving ECS interfaces. While these results are intuitive, the accuracy of the PMF calculations could be improved. Simulations of longer time scales will be needed to fully sample positional, orientational and conformational changes of proteins in all-atom simulations [55,56,57] in contrast to CG simulations [58,59,60,61,62,63].

Experimental studies support that protein-protein interactions may not be the same for all claudins. For example, there are significant differences in how claudin-5 and claudin-3 oligomerize and this is thought to be due to differences in key residues in TM3 [39]. To investigate the role of TM3 helices in the self-assembly of claudins in the membrane, Irudayanathan et al. [43] simulated the assembly of homology models of claudin-3 and claudin-5 in lipid bilayers at CG resolution. The self-assembly trajectory of claudin-3 monomers showed that the dimer with TM2–TM3 interface, detected in claudin-5 trajectories, was absent in claudin-3, while other dimer interfaces were detected. Mutating two key residues on the TM3 of claudin-5 as proposed by Rossa et al. [39] to those in claudin-3 (F139S/I142T) decreased the likelihood of TM2–TM3 dimerization in claudin-5 and vice versa. Mutating the same residues in claudin-3 to those in claudin-5 (S138F/T141I) resulted in a slight increase in dimerization probability of conformations similar to those that involve TM2–TM3. These simulations suggest that distortion of TM3 in claudin-3 prevents dimerization in the self-assembly process. While no significant changes were observed in the shape of strands in wild-type claudins compared to mutants, it is not true that all claudin protein-protein interactions result in the same strand structure. For example, recent X-ray crystallography of claudin-3 has shown that the TM3 helix is bent, in contrast to a straighter helical structure in claudin-4, -15 and -19 [64]. The bend in this helix is shown to affect the morphology of claudin strands.

CG simulations have also been used to study pore formation through *trans-*interactions between claudins in two parallel lipid bilayers. Irudayanathan et al. [43] performed CG simulations of 128 claudin-5 monomers in two adjacent lipid bilayers. 20 μs of self-assembly simulation showed instances of *trans-*interactions between the smaller ECS2 loop of monomers in adjacent membranes. Although these interactions were proposed previously [20,65], formation of pore-like structures requires more involved interactions, requiring longer simulation times.

Lipid environment is important in the regulation of claudin function [66,67] and it has been hypothesized to influence claudin oligomerization. Evidence suggests that removal of cholesterol can disrupt barrier function by altering the stability of tight junction complexes [68,69]. To investigate the influence of membrane composition on claudin oligomerization, Irudayanathan et al. [52] carried out simulations of claudin-5 monomers in lipid bilayers with different lengths of hydrophobic moieties (DLPC, DYPC, DPPC and POPC) and different cholesterol containing membranes (PC, PCS, DPC). Simulations suggest that the presence of cholesterol reduces the frequency of dimerization of claudins and, thus, the likelihood of strand formation in the lipid bilayers. In particular, the presence of cholesterol in the lipid bilayer prevented formation of dimers that involve interaction of TM helices [52]. The prevalent dimers observed in these simulations were those that involved limited interactions between ECS segments of claudin-5. The effect of claudin palmitoylation has also been studied using CG simulations but the study revealed no effect on strand formation [70]. However, cholesterol binding prevents dimerization of palmitoylated claudins, too.

The above CG studies have provided insight into claudin-claudin interactions and this type of model may improve our understanding of the factors which govern strand shape. However, time scales of computations are still too short to simulate dynamics of strand formation, even in CG simulations. Moreover, the limited resolution of CG simulations limits their ability to describe functional properties of proteins, such as ion transport, where channel function is controlled by specific and finely tuned interactions amongst proteins, ions and water.

## 7. Conclusions

Computational modeling and simulations have proven to be a very useful tool that have enabled researchers to combine much of what we have learned from numerous in vitro and in vivo studies of tight junctions in the past several decades. Recent advances in computational hardware and methodologies have permitted us to reach time scales and resolutions relevant for understanding claudin organization and function, ranging from atomistic simulations of ion permeation to CG simulations of claudin assembly to dynamic simulations of transport across multiple strands and Brownian dynamics simulations of ions across many channels. All of the models presented in this review have limitations and will need to be continually refined as more data becomes available. It will be an iterative process where modeling is continually compared with experimental data. As computing power increases and models become more accurate, it will become possible to expand simulations spatially and temporally to better understand the intersecting pathways which dynamically regulate tight junction structure and function. Such models will help us form and test new hypotheses, understand mechanisms of barrier dysfunction, discover means to increase or decrease tight junction barrier function and gain insight into approaches to promote trans-tight junction drug absorption.

## Abbreviations

The following abbreviations are used in this manuscript:

MD	Molecular Dynamics
CG	Coarse-grained
PMF	Potential of Mean Force
TER	Transepithelial Electrical Resistance
TJ	Tight Junction
TM	Transmembrane
ECS	Extracellular Segment
ECH	Extracellular Helix
FFEM	Freeze Fracture Electron Microscopy
